# Potency of Urea-Treated Halloysite Nanotubes for the Simultaneous Boosting of Mechanical Properties and Crystallization of Epoxidized Natural Rubber Composites

**DOI:** 10.3390/polym13183068

**Published:** 2021-09-11

**Authors:** Indra Surya, Kamaruddin Waesateh, Sitisaiyidah Saiwari, Hanafi Ismail, Nadras Othman, Nabil Hayeemasae

**Affiliations:** 1 Department of Chemical Engineering, Faculty of Engineering, Universitas Sumatera Utara, Medan 20155, Sumatera Utara, Indonesia; isurya@usu.ac.id; 2 Islamic Sciences Demonstration School, Prince of Songkla University, Pattani Campus, Pattani 94000, Thailand; qamarud@hotmail.com; 3 Research Unit of Advanced Elastomeric Materials and Innovations for BCG Economy (AEMI), Faculty of Science and Technology, Prince of Songkla University, Pattani Campus, Pattani 94000, Thailand; sitisaiyidah.s@psu.ac.th; 4 Department of Rubber Technology and Polymer Science, Faculty of Science and Technology, Prince of Songkla University, Pattani Campus, Pattani 94000, Thailand; 5 School of Materials and Mineral Resources Engineering, Engineering Campus, Universiti Sains Malaysia, Nibong Tebal, Penang 14300, Malaysia; ihanafi@usm.my (H.I.); srnadras@usm.my (N.O.)

**Keywords:** epoxidized natural rubber, halloysite nanotubes, urea, tensile properties, wide-angle X-ray scattering

## Abstract

Halloysite nanotubes (HNTs) are naturally occurring tubular clay made of aluminosilicate sheets rolled several times. HNT has been used to reinforce many rubbers. However, the narrow diameter of this configuration causes HNT to have poor interfacial contact with the rubber matrix. Therefore, increasing the distance between layers could improve interfacial contact with the matrix. In this work, Epoxidized Natural Rubber (ENR)/HNT was the focus. The HNT layer distance was successfully increased by a urea-mechanochemical process. Attachment of urea onto HNT was verified by FTIR, where new peaks appeared around 3505 cm^−1^ and 3396 cm^−1^, corresponding to urea’s functionalities. The intercalation of urea to the distance gallery of HNT was revealed by XRD. It was also found that the use of urea-treated HNT improved the modulus, tensile strength, and tear strength of the composites. This was clearly responsible for interactions between ENR and urea-treated HNT. It was further verified by observing the Payne effect. The value of the Payne effect was found to be reduced at 62.38% after using urea for treatment. As for the strain-induced crystallization (SIC) of the composites, the stress–strain curves correlated well with the results from synchrotron wide-angle X-ray scattering.

## 1. Introduction

Incorporation of fillers into rubber has been a major ingredient for rubber compounds. They are added to rubber for many purposes, such as improving durability, thermal stability, and even to cheapen the manufacturing costs [1]. So far, special awareness has been given to the addition of nano-fillers because they effectively boost the performance of rubber vulcanizate even at very low content. The factors affecting such enhancement are mainly due to the aspect ratio, degree of dispersion, and orientation of high aspect ratio filler particles [2]. Halloysite nanotubes (HNT) are a type of naturally occurring nano-filler composed of aluminum, silicon, oxygen, and hydrogen. Being an economically viable material, HNT has been available in many interesting research works; the novel properties and related applications of HNT have been successively reported over decades. For instance, the use of HNT as a viable nanoscale container in drug delivery, their usage as controlled release [3], as well as other specific applications, namely an ion adsorbent, ceramic materials, especially biocompatible implants, and as template for synthesis of rod-like nanoparticles [4].

HNT have recently been added in various types of rubber matrices [5,6]. However, HNT are not compatible with non-polar rubbers due to its polarity in nature [7,8] and are especially not compatible with natural rubber (NR). Scientists have been trying to solve this problem and improve their compatibility using several methods. These cover using silane coupling agents, optimizing the preparation methods, and modifying the chemical structure of rubber matrix. The last approach might be of great practical relevance to ensure the compatibility at any location along the polymer molecular chain [9,10]. In this work, an Epoxidized Natural Rubber (ENR) was introduced for preparing the composite to assure filler–matrix compatibility. However, there are still concerns regarding the characteristics of HNT itself. HNT is made of aluminosilicate kaolin sheets rolled several times. The narrow layer distance in such rolled sheet gives HNT poor interfacial contact with the rubber matrix during processing. Therefore, increasing the layer separation distance could improve the interfacial contact.

Recently, increasing the basal distance of the HNT layers has been one of the major approaches to improve the interfacial adhesion between HNT and rubber matrix [11]. Nicolini et al. [12] observed that urea can increase the basal spacing through a mechano-chemical process. It was reported that the basal distance shifted from 7.4 Å to 10.7 Å when the urea percentage increased and stabilized when the amount of urea approached 18%. It is claimed that the layers of HNT were intercalated by urea, making a material with a basal distance of 10.68 Å and with an expansion of 3.5 Å [13]. By increasing interlayer separation in HNT, easier intercalation of a polymer inside the HNT lumen and/or in HNT interlayer is assumed. This study only focused on the treatment of HNT itself without any inclusion of a polymer matrix.

Therefore, the aim of this study is to use urea-treated HNT in the ENR matrix to improve the overall properties of the composites. With this idea, the interfacial adhesion between HNT and ENR may be improved, while good rubber-filler interactions are assured by the polarity of ENR. The filler modification is expected to improve the compatibility and homogeneity of filler dispersion in the composites and to thereby enhance the reinforcing efficiency of HNT in filled ENR composites. This study proposed certain methods of evaluating the reinforcing efficiency of the composites, namely mechanical properties, dynamic properties, and SIC. The latter method is considered an interesting route, and not many reports have been focused on it. This can only be correlated for certain types of rubber, such as NR [14,15,16]. This is because NR has a very long chain that has an ability in arrangement and orientation for crystallization under stretching [17]. This ability to crystallize under strain is explained by the high regularity of molecular structure that is almost 100% of cis-1,4-polyisoprene [18].

Many studies of NR have been carried out together with in-situ deformation through in-situ X-ray diffraction techniques. Tosaka et al. [19] studied the effect of the different crosslink density on the SIC of vulcanized rubber, and they found that faster development in crystallinity was observed in the sample with higher crosslink density but at limited amounts. Toki et al. [20] indicated that the crystallinity increased with strain. They considered that stretched rubber can be differentiated into three phases: (i) an un-oriented amorphous phase, (ii) an oriented amorphous phase, and (iii) a crystalline phase. In ENR, the SIC lattice parameters of ENR were found to be crosslink independent regardless of quantity and method of crosslink. Higher crosslink density may induce faster SIC, and the volume of unit cell is larger than the reported values for NR, which is simply due to the epoxide group at the main chain [21].

SIC of unfilled and filled NR has also been focused by Poompradub et al. [22]; they found out that the onset strain for SIC decreased after adding filler. The degree of lattice deformation decreased with filler content, especially in the carbon black (CB)-filled composite. Chenal et al. [23] further explained that onset strain for SIC is ruled by the strain amplification induced in the presence of filler. Furthermore, different fillers behave different characteristics associating to the formation of rubber-filler interaction/reactions. This can be either accelerate or slow down SIC depending on NR matrix–chemical crosslink density. Similar observation was observed in vulcanized NR containing CB particles by Candau et al. [24].

From the reports above, the rubber–filler interactions may speed up the crystallization process at the certain network chain density. In this report, we presented parallel wide-angle X-ray scattering and tensile measurement on urea-treated HNT-filled ENR composites. To date, no reports have been revealed of detailed investigations regarding the relationship between mechanical and dynamic properties with SIC of rubber composites. The results explored from this work aim to give a scientific understanding of how the role of urea affects the overall properties of ENR/HNT composites and will be useful for manufacturing of rubber products based on ENR/HNT composites

## 2. Experimental Details

### 2.1. Materials

High ammonia centrifuged latex (HA) with 60% of dry rubber content (DRC) was used to prepare ENR. This latex was centrifuged and supplied by Chalong Latex Industry Co., Ltd., Songkhla, Thailand. The chemicals involved in the synthesis of ENR were Teric N30 as non-ionic surfactant, Formic acid and hydrogen peroxide for performic acid reaction were purchased from Sigma Aldrich (Thailand) Co. Ltd., Bangkok, Thailand. The HNT was mined and supplied by Imerys Ceramics Limited, Matauri Bay, New Zealand. HNT consists of the following components: SiO_2_ (49.5 wt%), Al_2_O_3_ (35.5 wt%), Fe_2_O_3_ (0.29 wt%), TiO_2_ (0.09 wt%), as well as CaO, MgO, K_2_O, and Na_2_O as traces. Stearic acid was supplied by Imperial Industrial Chemicals (Thailand) Co., Ltd., Bangkok, Thailand. ZnO was supplied by Global Chemical Co., Ltd., Samut Prakan, Thailand. N-cyclohexyl-2-benzothiazole sulfenamide was obtained from Flexsys America L.P., Akron, OH, USA, and soluble sulfur was purchased from Siam Chemical Industry Co., Ltd., Samut Prakan, Thailand.

### 2.2. Preparation of Epoxidized Natural Rubber

The synthesis of ENR was done by diluting DRC of latex to 15%. Next, 1 phr of non-ionic stabilizer (10% Teric N30) was added while stirring for 30 min at ambient temperature to expel the ammonia dissolved in HA. The epoxidation was performed using formic acid and hydrogen peroxide at 50 °C in a 10-L glass container under a stirring rate of 30 rpm. The total reaction time was fixed to obtain ENR with 20 mol% epoxide. The resulting ENR was coagulated with methanol followed by washing with water. Finally, it was dried in a vacuum oven at 50 °C prior to use.

### 2.3. Preparation of Urea-Treated HNT

As for the urea-treated HNT, it was prepared according to Nicolini et al. [12]. The contents of urea varied among 10%, 14%, 18%, and 20% of HNT. Later, the samples were labeled as ENR20 for untreated HNT and as ENR20U10–ENR20U20 for the urea contents from 10% to 20%, respectively. The intercalation of urea into the interlayer of HNT was enabled mechano-chemically. Firstly, HNT was mixed with the urea and ground by ball milling in a ceramic container. The urea-treated HNT was purified by washing with isopropanol and dried in an oven at 70 °C for 12 h. The urea-treated HNT was finally ground in a mortar prior to use in compounding.

### 2.4. Preparation of ENR/HNT Composites

The recipe for the preparation of ENR/HNT composites is given in Table 1. ENR with 20 mole% epoxide was compounded with HNT (e.g., untreated and urea-treated HNT depending on the formulation) and other ingredients except for the curatives (CBS and sulfur) in a Brabender plasticorder. The initial mixing temperature was set at 50 °C with a rotor speed of 60 rpm. The compound was then sheeted on a two-roll mill while the curatives were incorporated. Finally, samples of the various compounds were later tested for curing characteristics.

### 2.5. Measurement of Curing Characteristics

The curing properties of the composites were measured according to ASTM D5289 using a moving die rheometer (Rheoline, Mini MDR Lite, Prescott Instruments Ltd., Tewkesbury, UK). The operating temperature was set at 150 °C. The data in terms of torques, scorch time (t_s2_), and curing time (t_c90_) were recorded. The t_s2_ and t_c90_ were used in calculating the curing rate index (CRI) as follows:

(1) $$\mathrm{CRI}=\frac{100}{t_{c90}-t_{s2}}$$

### 2.6. Fourier Transform Infrared-Spectroscopic Analysis (FT-IR)

Attachment of urea onto HNT was confirmed by Fourier transform infrared-spectroscopy (FTIR) using FTIR spectroscope model TENSOR27 (Bruker Corporation, Billerica, Massachusetts, USA). The spectra were recorded in transmission mode with a 4 cm^−1^ resolution over 4000–550 cm^−1^.

### 2.7. X-ray Diffraction Analysis (XRD)

The XRD analysis of pure HNT and urea-treated HNT was carried out by using PHILIPS X’Pert MPD (Eindhoven, Netherlands) with CuKα radiation (λ = 0.154 nm) at 40 kV and a current of 30 mA and Bruker D2 Phaser (Billerica, MA, USA) with CuKα radiation source (λ = 0.154 nm) and a current of 10 mA. The diffraction patterns were scanned in the diffraction angles 2θ of 5–30° with a step size of 0.05° and 3°/min scan speed. The d-spacing of HNT layers in particles was estimated from Bragg’s equation.

### 2.8. Measurement of Mechanical Properties and Hardness

Measurement of tensile properties was done according to ASTM D412. The sample’s dimension was based on Die C dumbbell shape. The test was performed using a universal testing machine (Tinius Olsen, H10KS, Tinius Olsen Ltd., Surrey, UK) at a crosshead speed of 500 mm/min. The determinations recorded were the modulus at 100% (M100) and 300% (M300) strains, tensile strength, and elongation at break. The tear strength of the respective composites was tested using the same machine and crosshead speed according ASTM D624. A type C (right angle) test piece was selected for experiment. The last measurement was the hardness property of the samples. It was performed according to ASTM D2240 using a Shore A type manual durometer.

### 2.9. Determination of Crosslink Density

The crosslink density of the composite was determined by equilibrium swelling method as described in ASTM D6814. The specimens were cut into a circular shape and weighed before and after immersing in toluene for 72 h. The modified Flory–Rehner equation was implemented for calculating the cross-link density (*υ*) [25]:

(2) $$\nu =\frac{1}{{2M}_{c}}$$

(3) Mc=ρ⋅V0⋅(Vr13−Vr2)ln(1−Vr)+Vr+μ⋅Vr2

where *M_c_* is the number-average molecular weight of the rubber chains between crosslinks, *µ* is the parameter for rubber-toluene interactions (*µ* = 0.42), *ρ* is the bulk density of the specimen, *V*_0_ is the molar volume of the toluene (*V*_0_ = 106.2 cm^3^/mol), and *V_r_* is the volume fraction in the swollen specimen, defined as follows [26]:

(4) $$V_{r}=\frac{\left(D-FT\right)\cdot \rho^{-1}}{\left(D-FT\right)\cdot \rho^{-1}+A_{0}\cdot \rho_{s}^{-1}}$$

where *T* is the weight of the specimen, *D* is the weight of the de-swollen specimen, *F* is the weight fraction of the insoluble parts, *A*_0_ is the weight of the toluene absorbed in swollen specimen, *ρ* is the density of the specimen, and *ρ_s_* is the density of the toluene (0.886 g/cm^3^).

### 2.10. Scanning Electron Microscopy

The freshly fractured surfaces of samples from tensile testing were used to observe the dispersion of untreated and urea-treated HNT in the rubber matrix. The morphology was captured using a scanning electron microscope (SEM; FEI Quanta FEG 400, Thermo Fisher Scientific, Waltham, MA, USA). A layer of gold/palladium coated the specimen to eliminate charge built during imaging.

### 2.11. Dynamic Properties

The dynamic properties of the composites were analyzed using a Rubber Process Analyzer (RPA), model D-RPA 3000 (MonTech Werkstoffprüfmaschinen GmbH, Buchen, Germany). At first, the tested samples were cured at 150 °C based on the t_c90_ tested by the same RPA. The samples were then cooled down to 60 °C. At this time, at 10-Hz fixed frequency, the strain was varied from 0.5 to 90%. This was to determine the storage modulus (*G′*) as function of strain in the composites. The raw *G′* record was further used to study the filler–filler interactions via a so-called Payne effect. The Payne effect was calculated as follows:

Payne effect = *G′_I_* − *G′_f_* (5)

where *G′_i_* and *G′_f_* were the *G′* at 0.5% and 90% strains, respectively. A larger Payne effect indicates weaker rubber–filler interactions.

### 2.12. Wide-Angle X-ray Scattering

The SIC of the composite was correlated with the stress-strain curves of the composite. SIC and other related results were obtained from a synchrotron wide-angle X-ray scattering (WAXS) analysis. The experiment was carried out using Beamline 1.3 W at the Siam Photon Laboratory, Synchrotron Light Research Institute (SLRI), Nakhon Ratchasima, Thailand. The distance between sample and detector was 115.34 mm, measured using a wavelength of 0.138 nm. A CCD detector (Rayonix, SX165, Rayonix, L.L.C., Evanston, IL, USA) with a diameter of 165 mm was equipped to capture the WAXS profile. The scattering angle was calibrated using 4-Bromobenzoic acid as a standard material.

Prior to the testing, a Die C type dumbbell specimen was held on the grips of a stretching apparatus. The sample was stretched at a crosshead speed of 50 mm/min to a given strain and was then relaxed in the deformed state for 30 s. WAXS was recorded and stretching then continued to the next predetermined strain until the characterization was complete. The degree of crystallinity (*X_c_*) was calculated based on the data obtained from the WAXS profiles using the following equation:

(6) $$\mathrm{Degree}\;\mathrm{of}\;\mathrm{crystallinity}\;(X_{c})=\left(\frac{A_{c}}{A_{c}+A_{a}}\right)\times 100$$

where *A_c_* is and *A_a_* are the areas under the crystalline peak of interest and the amorphous halo, respectively.

The orientation parameter (OP) was also determined from the Hermann equation, as follows:

(7) $$\mathrm{OP}=\frac{{3[\cos}^{2}\varphi ]-1}{2}$$

where *φ* is the azimuthal angle related to the direction of strain. The mean value of *cos*^2^ *φ* is calculated as follows:

(8) $${[\cos}^{2}\varphi ]=\frac{\int_{0}^{\pi }I_{c}(\varphi )\cdot \cos^{2}\varphi \cdot \sin\varphi \cdot d\varphi }{\int_{0}^{\pi }I_{c}(\varphi )\cdot \sin\varphi \cdot d\varphi }$$

where *I_c_* (*φ*) is the scattering intensity of the crystal at *φ*. *I_c_* (*φ*) is normalized by subtracting the minimum scattering intensity of the amorphous component of the original WAXS intensity [27,28].

## 3. Results and Discussion

### 3.1. Curing Characteristics

**Figure 1:**
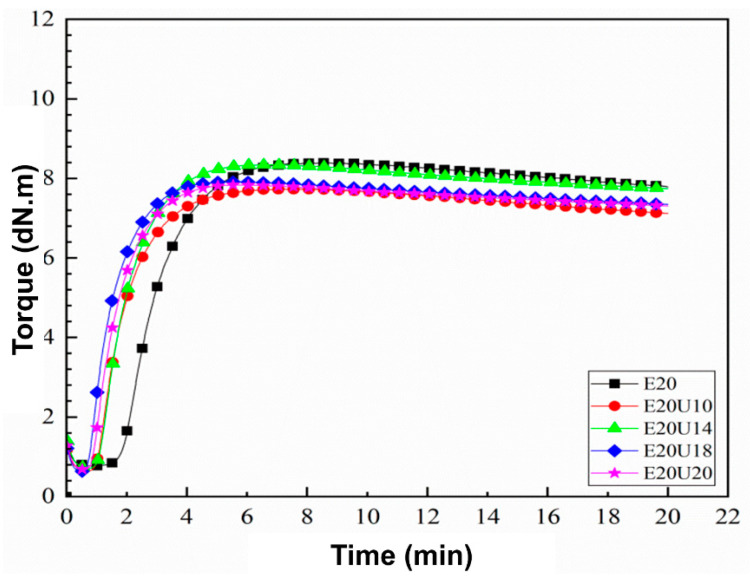
Curing characteristic curves of ENR/HNT composites filled with untreated and urea-treated HNT.

Curing curves of the composites in the presence of untreated and urea-treated HNT are shown in Figure 1. The raw data are summarized in Table 2. It was found that the scorch time (t_s2_) and curing time (t_c90_) decreased, while CRI increased with urea content. Theoretically, an alkaline chemical substance relatively accelerates the vulcanization process, while an acidic compound would retard it. Urea is an amine substance with alkaline characteristics [29,30]. Therefore, increasing urea content contributed to the rate of vulcanization.

It was observed that the use of urea-treated HNT slightly changed in M_L_, while the M_H_ increased to its maximum at 14% of urea. This increase in M_H_ of the composites was attributed to the interactions of HNT and ENR with urea. Above 14% urea content, M_H_ decreased again, which can be attributed to the plasticizing effects of the urea. Similar observation was made regarding the delta torque (M_H_–M_L_), indicating that urea plays an important role in these composites.

### 3.2. FT-IR Analysis

**Figure 2:**
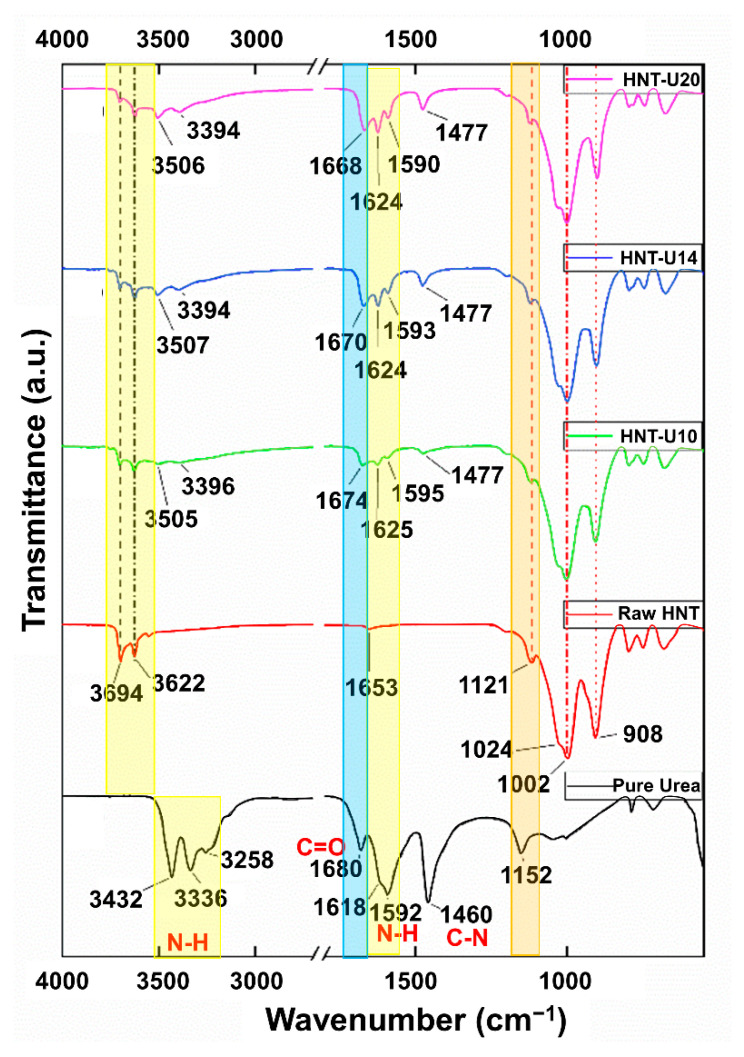
Infrared spectra of pure urea and untreated and urea-treated HNT.

The FTIR spectra of urea, raw HNT, and urea-treated HNT are given in Figure 2. For the pure urea, peak absorption at 3432 cm^−1^ is assigned to N-H out-of-plane stretching vibrations and the bands at 3336 cm^−1^ and 3258 cm^−1^ are formed by N-H in-plane stretching vibrations. The N-H bending vibrations are also absorbing at 1619 cm^−1^ and 1592 cm^−1^. The peak at 1460 cm^−1^ is assigned to the stretching vibrations of C-N, while the NH_2_ rocking vibrations give absorption at 1152 cm^−1^ [31]. This clearly corresponds to the chemical structure of urea. The increased transmittance in the ranges 3500–3300 cm^−1^ and 1800–1400 cm^−1^ was clear as the level of urea was increased, corresponding to the reference band of pure urea. This corroborates that the urea was attached on the surfaces of HNT. The inner surface OH was represented at 3694 cm^−1^ and 3622 cm^−1^.

To ensure that intercalation of urea to HNT was happening, the spectrum in the range 1800–1400 cm^−1^ was considered. Basically, the amine group available in urea can form hydrogen bonds with the hydroxyl groups on HNT. This could have caused the an amine band from 1618 cm^−1^ for pure urea to 1625 cm^−1^, 1624 cm^−1^, and 1624 cm^−1^ for urea-treated HNT. This happens together with the appearance of new peaks at around 3505 cm^−1^ and 3396 cm^−1^ [8] and also with shifting of carbonyl peak at 1680 cm^−1^ to 1674 cm^−1^, 1670 cm^−1^, and 1668 cm^−1^ [32]. Such findings are in good agreement with the report of Makó et al. [33], showing that the kaolinite–urea complex was formed after modification. Since HNT is chemically similar to clay, its interaction mechanisms with urea are expected to be similar.

**Figure 3:**
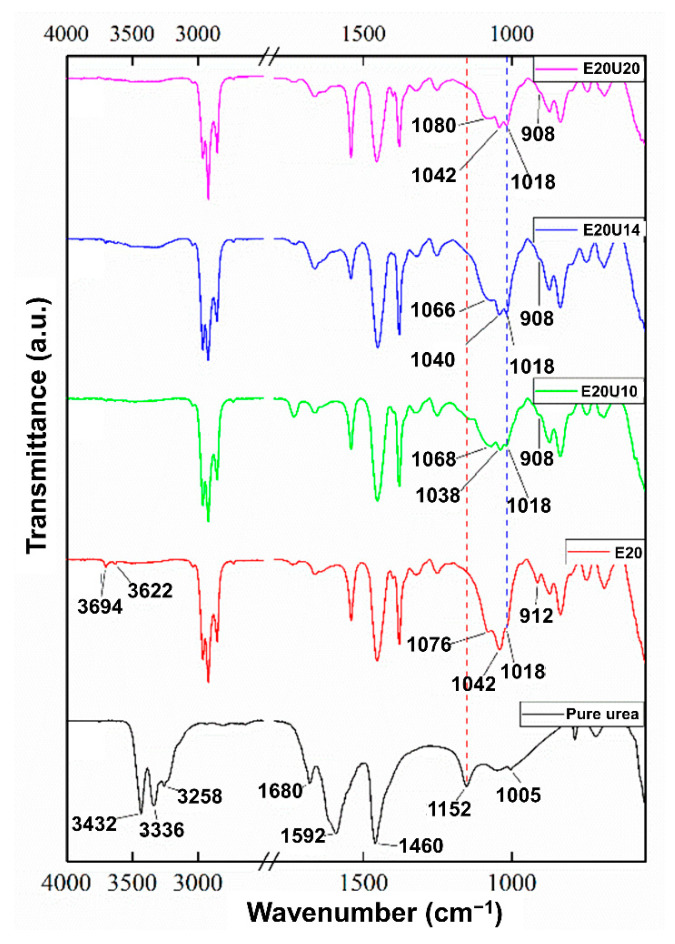
Infrared spectra of ENR/HNT composites in the presence of untreated and urea-treated HNT.

The FTIR spectra of the ENR composites filled with untreated and urea-treated HNT are shown in Figure 3. The hydrocarbon characteristics absorption peaks at 1662 cm^−1^, 1448 cm^−1^, 1375 cm^−1^, and 837 cm^−1^ relate to the stretching vibrations of C=C bonds, bending vibrations of CH_2_ and CH_3_ groups, and out-of-plane deformations of =C-H group, respectively. The epoxide ring absorption took place at 873 cm^−1^ and 1250 cm^−1^. Interestingly, the absorption at 3694 cm^−1^ and 3622 cm^−1^ was assigned to inner surface OH, and the outer OH groups are reduced with increasing urea contents [34,35]. Broadened shoulder peak at 1152 cm^−1^ and weakened peak absorption at 3694 cm^−1^, 3622 cm^−1^, and 912 cm^−1^ were also found. This is a clear indication that the treatment of HNT with urea reduced transmittance of the inner surface hydroxyl groups on HNT, the interactions formed between HNT and urea. It is worth noting that the location of the Si–O stretching in the region of 1100–1000 cm^−1^ was shifted with the addition of urea. This suggests that ENR interacted with the surfaces of HNT through its epoxide groups, making hydrogen bonds with hydrogen attached to electronegative N atom in urea-treated HNT [36].

### 3.3. X-ray Diffraction Analysis

**Figure 4:**
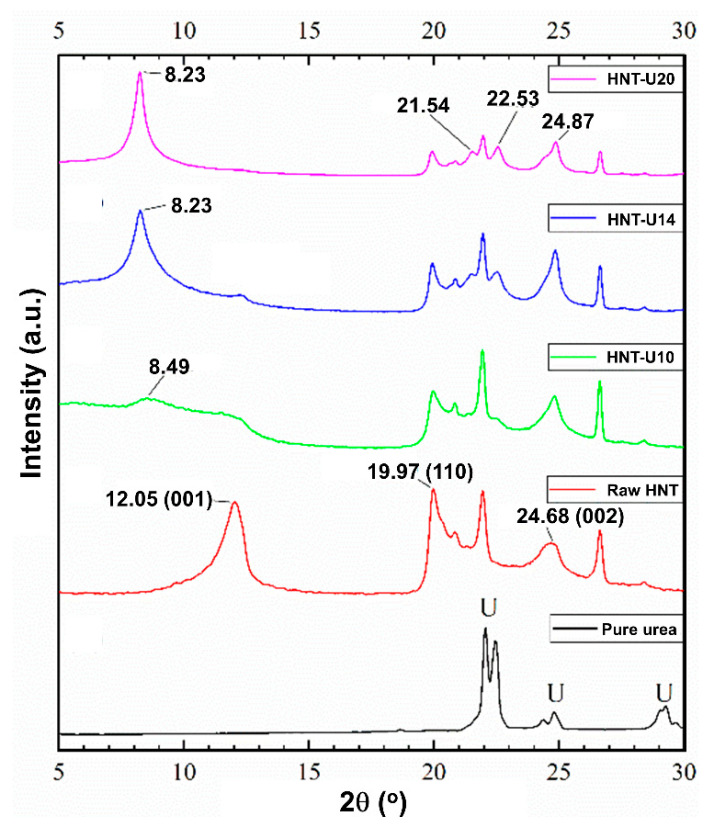
XRD scattering patterns of pure urea and untreated and urea-treated HNT.

**Figure 5:**
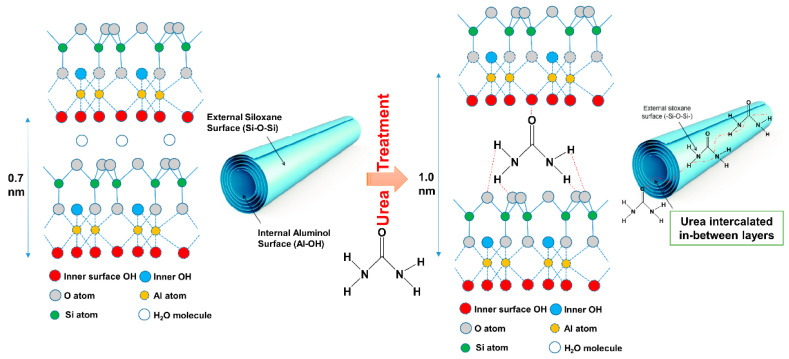
Proposed model of intercalation and interactions of urea with HNT via mechano-chemical processing modified from Yuan et al. [37].

The XRD patterns of the untreated and urea-treated HNT are shown in Figure 4. The untreated HNT shows a 001 reflection at 2θ of 12.05°, indicating basal distance of 7.33 Ǻ, which is characteristic of dehydrated Halloysite. This peak tended to disappear as the amount of urea was increased. All the HNT crystals were fully intercalated with urea, resulting an increase in basal distance from 7.33 Ǻ (2θ = 12.05°) to 10.72 Ǻ (2θ of 8.49° and 8.23°). This is verified by shifting of the 001 peak from 2θ of 12.05° to 8.49° and 8.23°. One important feature of intercalated HNT is the development of two peaks at 21.54° and 22.53° in the range with saw tooth diffraction peaks. These may be associated with decreased lattice strain and/or increased crystallite size [33]. A schematic model proposed for the interactions in urea-treated HNT is shown in Figure 5. This scheme demonstrates an increase of basal distance between layers of HNT due to the penetration of urea; hydrogen bonds are formed between the hydroxyl groups of HNT and amine contained in urea.

**Figure 6:**
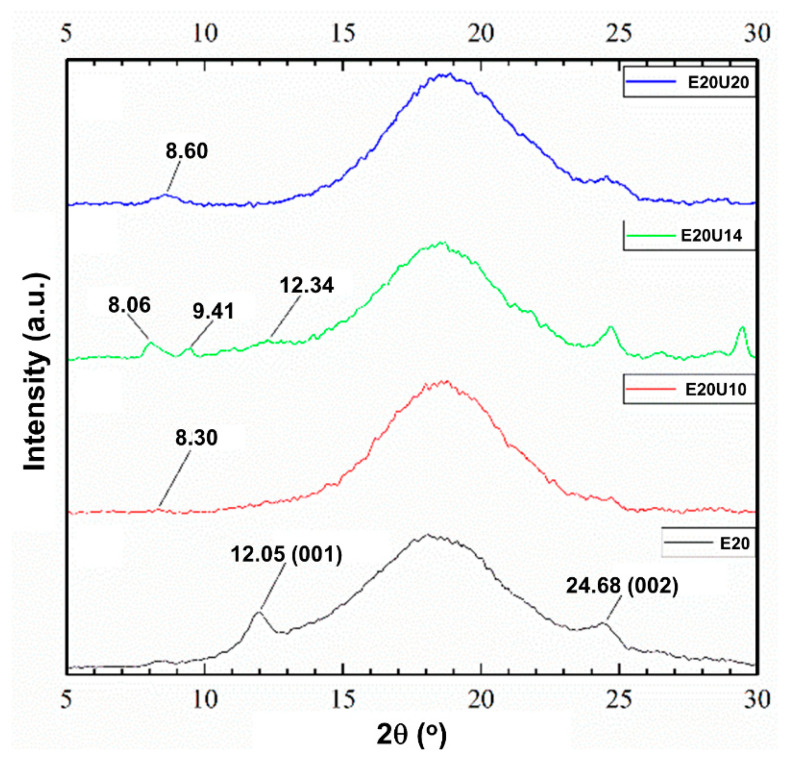
XRD scattering patterns of ENR/HNT composites filled with untreated and urea-treated HNT (un-deformed specimen).

Figure 6 shows the XRD patterns of the ENR composites filled with untreated and urea-treated HNT. The diffraction peak at 2θ of 12.05° (d is 7.33 Ǻ) related to 001 plane and is absent after urea-treatment of HNT. This peak is shifted to the lower 2θ at 8.30°, 8.06°, 9.41°, or 8.60°, which correspond to basal gallery sizes of 10.64 Ǻ, 10.95 Ǻ, 9.38 Ǻ, and 10.27 Ǻ, respectively. The modification of HNT by mechano-chemical processing altered the crystal lattice of HNT. The modification with urea of HNT increased the basal distance, and hence, a lesser 2θ is presented due to the intercalation of ENR molecules to the gallery of HNT [38,39]. Once the intercalation occurred, possibly there were also further interactions between ENR and HNT in the system.

### 3.4. Dynamic Properties

**Figure 7:**
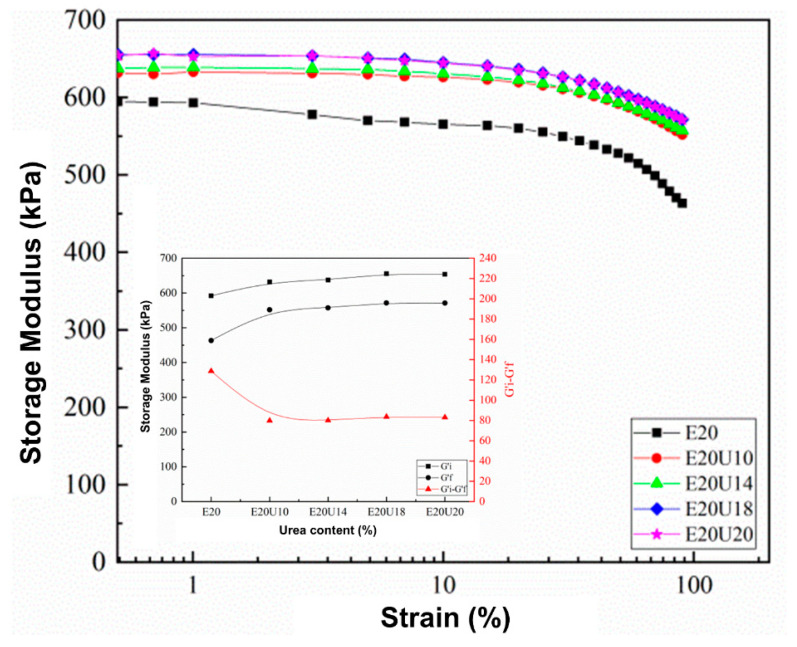
Strain dependence of storage modulus and Payne effect in ENR/HNT composites filled with untreated and urea-treated HNT.

One approach to assessing possible interactions in a composite is by use of the dynamic properties. In this study, storage modulus and the Payne effect of the ENR/HNT composites made with untreated or urea-treated HNT filler were analyzed to assess the rubber–filler interactions. The results are presented in Figure 7. The storage modulus (*G′*) of the composites was constant in the low strain region but slightly decreased with strains larger than 50%. This is common for a viscoelastic material and is due to the molecular stability of rubber. It is noticeable that the *G′* increased with urea content, indicating interactions between urea-treated HNT with ENR. There are two factors determining the increase in *G′*, namely the dispersion of HNT facilitated by urea as well as improved interfacial interactions between HNT and ENR caused by urea-treatment of the HNT. The Payne effect relates to the rubber–filler interactions in the composite, and its measure here was the difference in *G′* between low and high strains [40]. A higher Payne effect indicates lesser rubber–filler interactions. It was found that the Payne effect decreased with urea content, where the value of Payne effect was found to be reduced at 62.38% after using urea for treatment. This might be due to stable rubber–filler interactions of urea-treated HNT filler in the ENR matrix. The results on the dynamic properties confirm interactions between urea-treated HNT and ENR, supporting the previous FTIR and XRD.

### 3.5. Mechanical Properties

**Figure 8:**
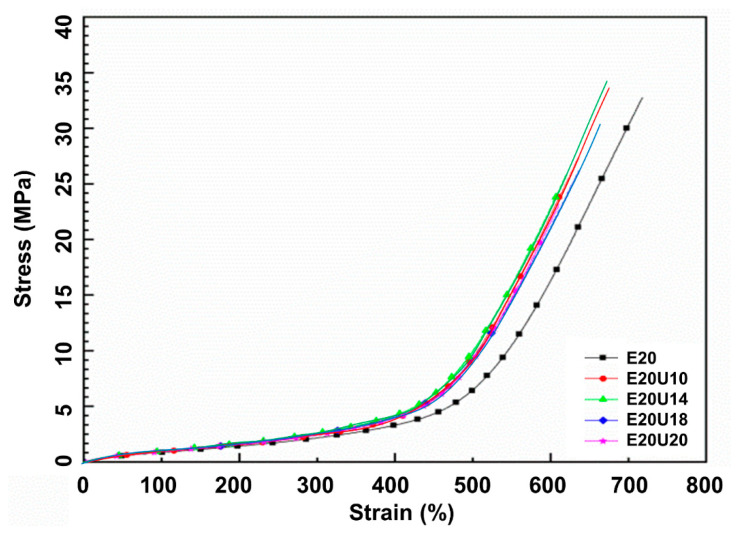
Stress–strain dependence of ENR/HNT composites filled with untreated and urea-treated HNT.

Figure 8 shows the stress–strain curves of the ENR composites made with untreated and urea-treated HNT. The stress–strain curves show the SIC. Higher stress response was found for the composites filled with urea-treated HNT, suggesting that the samples became stronger after urea-treated HNT was used. Higher compatibility between ENR and HNT in the presence of urea is responsible for these findings. Further, the area underneath the stress–strain curve was examined to confirm the compatibility of rubber and the filler. This indicates the toughness of a material [41]. Larger area underneath the curve corresponds to the greater toughness. The urea-treated HNT composites showed a greater area underneath the stress–strain curve than the untreated counterpart and therefore greater toughness. The curves shown are further discussed and regards crystallization behavior.

The mechanical properties, such as modulus, tensile strength, elongation at break, tear strength, and hardness, are shown and summarized in Table 3. It was found that the modification of HNT with urea via mechano-chemical process led to increase in the tensile and tear strength of the composites. The tensile strength with untreated HNT was 33.67 MPa and increased up to 35.15 MPa at 14% of urea content. The tear strength of reference sample increased from 38.29 N/mm to 38.36, 39.24, 37.42, and 35.60 N/mm. Using urea-treated HNT has evidently proven that an intercalation had taken place. The evidence for such boosting has already been shown by the previous sessions. The reduction of tensile and tear strength at urea content over 14% might be due to some destruction of the HNT structure that occurred during mechano-chemical process, as seen in SEM images. The significant change in the rubber–filler interaction of ENR and HNT can be also verified from the stresses at 100% (M100) and 300% (M300) strains (see Table 3). It can be seen that the M100 and M300 increased over the urea content. As the higher urea was introduced to the rubber, the greater interactions occurred, resulting in harder and stiffer composites. Such finding is clearer when examining the M300. The result obtained here corresponded well with the reduction of elongation at break of the composites. The reduction of elongation at break was found due to lower flexibility of molecular chain and contributed to the increase of interaction point in the composites. Similar observation for the modulus was also encountered for the hardness property.

### 3.6. Morphological Properties

**Figure 9:**
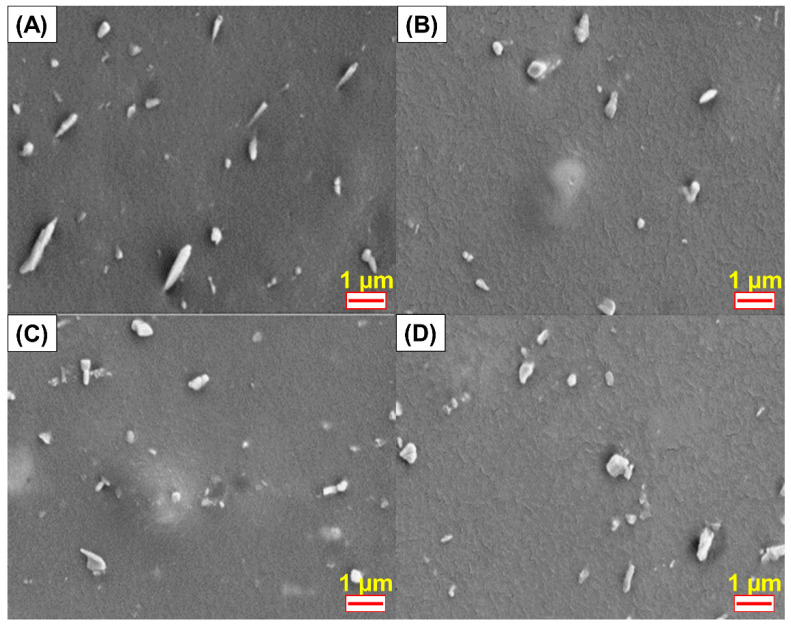
SEM photographs of ENR/HNT composites filled with untreated and urea-treated HNT; E20 (**A**), E20U10 (**B**), E20U14 (**C**), and E20U20 (**D**).

The dispersion of HNT within the rubber matrix can be assessed from the SEM images shown in Figure 9. Good dispersion of urea-treated HNT was observed with but shortened lengths of the dispersed HNT particles in the matrix are due to the destruction of HNT structure. This is in agreement with the decreased tensile strength and tear strength observed for comparatively high urea contents. Similar observations were previously reported for micro-fractured surfaces in NR composites having other fillers in the presence of a compatibilizer [42].

### 3.7. Wide-Angle X-ray Scattering

**Figure 10:**
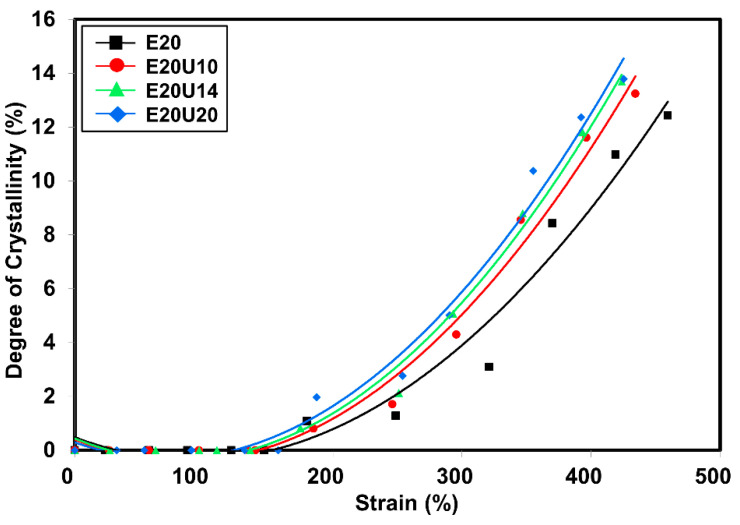
Strain dependence on degree of crystallinity of ENR/HNT composites filled with untreated and urea-treated HNT.

**Figure 11:**
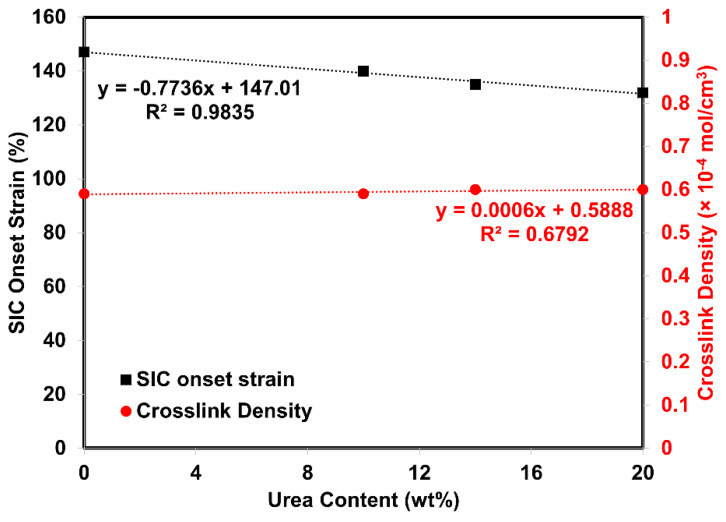
SIC onset strain (primary axis) and crosslink density of ENR/HNT composites filled with untreated and urea-treated HNT.

In the section on mechanical properties, the stress–strain behavior of the composites was associated with SIC. Since the nominal strain rate for tensile measurement and SIC is not similar (e.g., 0.42 s^−1^ and 0.042 s^−1^ for tensile test and WAXS, respectively). The correlation was made in the view of the stress versus crystallinity only. Previously, it was clear that the treatment of HNT by urea influenced the mechanical properties. The main factor is definitely the improved compatibility between ENR matrix and urea-treated HNT filler. The degree of crystallinity (*X_C_*) versus strain deformation is shown in Figure 10. Crystallinity was estimated from the areas in diffraction pattern for 200 and 120 plane reflections [43,44]. The *X_C_* increased with strain due to molecular chain orientation, as expected. The onset strain for SIC was determined from interception of a regression line for *X_c_* as a function of strain (see Figure 11). The onset for SIC with urea-treated HNT filler was observed to be lower as a function of urea content. The interaction that takes place in the presence of urea can help to pull the surrounding molecular chains and speeds up the crystallization process. When considering the *X_C_* and the stress propagation from tensile measurement (see Figure 8), it is clear that the *X_C_* corresponded well with the stress, and the trend of the curves is also similar as urea content increased. From the stress–strain curves, it is obvious that the stress started to increase towards the urea content. This is responsible to the formation of interfacial contact, as discussed earlier.

Earlier onset for SIC can be found usually for the filled composite. Poompradu et al. [22] reported that the lateral crystallite size was decreased, but the orientational fluctuation increased by the inclusion of filler. The lattice of the SIC changed almost linearly with the nominal stress. In addition, the degree of lattice deformation decreased with the filler content, especially in the CB-filled system. In addition to this, onset for SIC was differently observed depending on the filler’s characteristics. Ozbas et al. [45] compared the SIC of graphene and CB-filled composites. They found that the onset of SIC occurs at significantly lower strains for graphene-filled NR samples compared with CB-filled NR even at low loadings. Chenal et al. [23] further explained that the onset of SIC is ruled by the strain amplification induced by the filler presence. Moreover, additional interaction in rubber network is also responsible for either accelerating or slowing down the crystallization rate depending on rubber matrix chemical crosslink density. Candau et al. [24] together with the report of Ozbas et al. [45] further emphasized that the rubber–filler interactions may fasten the SIC at low crosslink density. This is because high crosslinking may interfere the chain orientation and results in reduction of the SIC. Therefore, the crosslink density of this composite was also reported and shown in Figure 11. It can be seen that the crosslink density observed was more or less the same over the content of urea. This is a good indication that network chain density is unchangeably involved in the development of SIC regardless of urea content. As a consequence, it can be said that the change in SIC was promoted by rubber–filler interactions.

**Figure 12:**
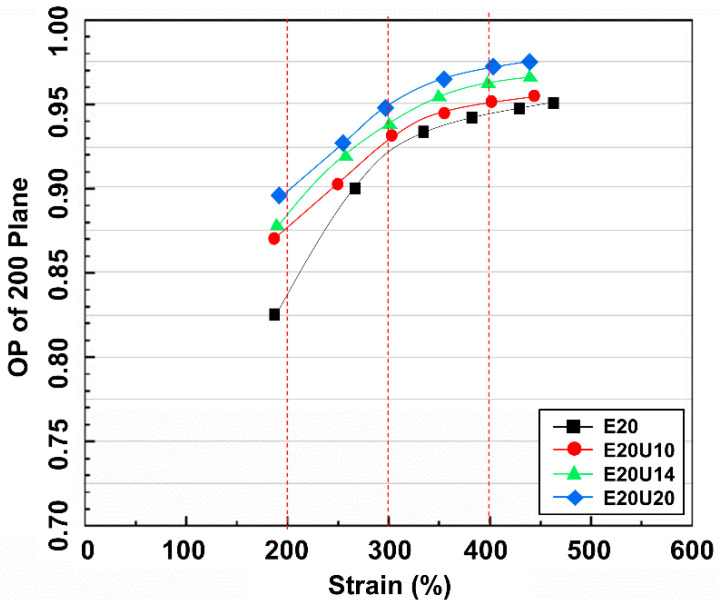
Strain dependence on orientation parameter of ENR/HNT composites filled with untreated and urea-treated HNT.

The orientation parameter (OP) indicates indirectly the molecular chain orientation and alignment and can be calculated from the Herman equation [27,28]. The OP for the composites is shown in Figure 12. Completely oriented molecular chains would give OP value 1 [46]. Here, the OP for the composites was lesser at low strains and grew with increasing strain, confirming that stretching oriented the molecular chains. The composites filled with urea-treated HNT showed higher OP values at low strains, indicating that the modification of HNT increases rubber–filler interactions, and accordingly, more molecular chain orientation was found for composites with urea-treated HNT.

**Figure 13:**
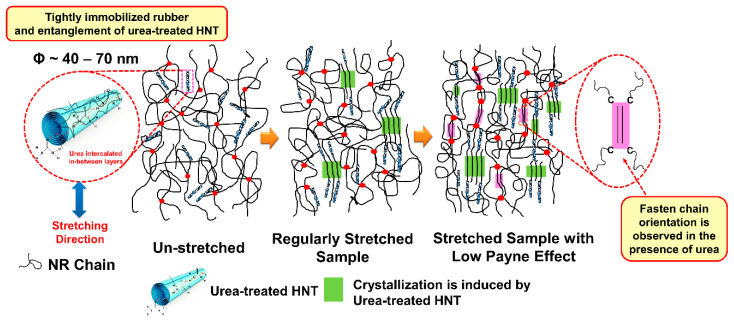
Schematic model representing the crystallization development of the ENR/HNT composites filled with urea-treated HNT.

From these results, correlation between the SIC and corresponding interaction between ENR and HNT in the presence of urea is represented in a schematic model shown in Figure 13. Referring to the scheme, nothing happened at the un-stretched stage of the sample; the ENR matrix may be in contact with the HNT due to the interfacial interaction created by the unique characteristics of the HNT. When the strain was applied to the sample, crystallization of the ENR was then induced due to the stress concentration point on the HNT surfaces, and the crystallinity increased in association with the orientation of the HNT. HNT is orientated and aligned along the stretching direction. As a consequence, the ENR chains rearrange and crystallize. This always happens regardless of whether untreated or urea-treated HNT is concerned. This kind of phenomenon usually occurs in the filled composites, and it has been reported elsewhere [23,24,45]. However, it is interesting to highlight that the crystallinity of the ENR matrix increases steeply due to the collaborative crystallization of ENR and HNT in association with the contribution of the urea. Higher rubber–filler interactions as indicated by lower Payne effect is responsible for such change. The presence of the urea has played an important role in pulling the surrounding molecular chains. Thus, a significant increase in crystallization is observed at higher strains, and this is in agreement with results observed previously in the stress–strain behaviors and WAXS profiles.

## 4. Conclusions

Urea-treated HNT filler was successfully prepared via a mechano-chemical process. Attachment of urea on HNT was verified by FTIR, where new peaks appeared around 3505 cm^−1^ and 3396 cm^−1^, corresponding to urea’s functionalities. The intercalation of urea in the gallery of HNT was revealed by XRD results. The development of two peaks at 21.54° and 22.53° in the range of saw tooth diffraction peak and the shifting of the peak at 2θ of 12.05° to 8.23° correlated to the decrease of the lattice strain and/or to the increment of the crystallite size. The t_s2_ and t_c90_ decreased with urea content due to alkaline nature of urea. The M_H_ was optimal at 14% urea content. Increased tensile strength and tear strength were also observed due to the improved filler–matrix interfacial adhesions of HNT with the rubber matrix. This is confirmed by the dynamic properties of the composites, as the value of Payne effect was clearly reduced to 62.38% after using urea for treatment. The SIC in the composites exhibited a clear change, as the strain upturn was an earlier strain during stretching, indicating faster crystallization caused by better interfacial interactions within the composites. The *X_c_* was directly observed with XRD during stretching, and it corresponded well with the tensile moduli of the composites. It is noted that correlation between SIC and mechanical strength may not be enough; an alternative testing, such as a fatigue test, is recommended in future work.

Based on the observations overall, it can be concluded that a urea content of about 14% in HNT filler is highly recommended for preparing composites with ENR matrix to ensure the compatibility and strength of the composite. On top of that, the use of urea can be the solution of choice for improving the interaction between ENR and HNT. It can offer improvements in processing behavior without the need to use complicated and costly silane coupling-agent systems.

## Figures and Tables

**Table 1:** Formulation of ENR composites filled with untreated and urea-treated HNT.

Raw Material	Amount (phr)
ENR	100.0
Stearic acid	1.0
Zinc oxide	5.0
HNT *	5.0
CBS	2.0
Sulfur	2.0

Remark: * HNT was treated with various content of urea.

**Table 2:** Scorch time (t_s2_), cure time (t_c90_), minimum torque (M_L_), maximum torque (M_H_), delta torque (M_H_–M_L_), and CRI for the ENR/HNT composites made with untreated or urea-treated HNT.

Sample	t_s2_ (min)	t_c90_ (min)	M_L_ (dN.m)	M_H_ (dN.m)	M_H_–M_L_ (dN.m)	CRI (min^−1^)
E20	2.29	4.75	0.76	8.40	7.64	40.65
E20U10	1.38	3.51	0.71	7.92	7.21	46.95
E20U14	1.42	3.52	0.68	8.35	7.67	47.62
E20U18	1.02	2.82	0.64	7.84	7.20	55.56
E20U20	1.18	3.06	0.63	7.74	7.11	53.19

**Table 3:** Modulus at 100% (M100), 300% (M300), tensile strength (TS), elongation at break (EB), tear strength (Ts), and hardness of ENR/HNT composites filled with untreated and urea-treated HNT.

Sample	M100 (MPa)	M300 (MPa)	TS (MPa)	EB (%)	Ts (N/mm)	Hardness (Shore A)
E20	0.86 ± 0.03	2.25 ± 0.03	33.67 ± 1.61	717 ± 10	38.29 ± 0.94	39.3 ± 0.3
E20U10	0.89 ± 0.04	2.34 ± 0.23	34.95 ± 0.51	660 ± 49	38.36 ± 0.51	41.6 ± 0.4
E20U14	0.95 ± 0.05	2.58 ± 0.18	35.15 ± 0.42	627 ± 28	39.24 ± 0.54	42.2 ± 0.4
E20U18	0.96 ± 0.01	2.59 ± 0.07	30.59 ± 1.22	618 ± 20	37.42 ± 0.72	42.7 ± 0.3
E20U20	0.97 ± 0.03	2.63 ± 0.03	26.87 ± 1.11	615 ± 13	35.60 ± 0.50	43.9 ± 0.7

## Data Availability

The data presented in this study are available on request from the corresponding author.

## References

[1] Arrighi V., McEwen I., Qian H., Prieto M.S. (2003). The glass transition and interfacial layer in styrene-butadiene rubber containing silica nanofiller. Polymer.

[2] Ismail H., Pasbakhsh P., Fauzi M.A., Bakar A.A. (2008). Morphological, thermal and tensile properties of halloysite nanotubes filled ethylene propylene diene monomer (EPDM) nanocomposites. Polym. Test..

[3] Price R.R., Gaber B.P., Lvov Y. (2001). In-vitro release characteristics of tetracycline HCl, khellin and nicotinamide adenine dineculeotide from halloysite; a cylindrical mineral. J. Microencapsul..

[4] Du M.L., Guo B.C., Jia D.M. (2006). Thermal stability and flame retardant effects of halloysite nanotubes on poly(propylene). Eur. Polym. J..

[5] Jia Z., Luo Y., Guo B., Yang B., Du M., Jia D. (2009). Reinforcing and flame-retardant effects of halloysite nanotubes on LLDPE. Polym. Plast. Technol. Eng..

[6] Vahedi V., Pasbakhsh P., Chai S.-P. (2015). Toward high performance epoxy/halloysite nanocomposites: New insights based on rheological, curing, and impact properties. Mater. Des..

[7] Rooj S., Das A., Thakur V., Mahaling R., Bhowmick A.K., Heinrich G. (2010). Preparation and properties of natural nanocomposites based on natural rubber and naturally occurring halloysite nanotubes. Mater. Des..

[8] Paran S., Naderi G., Ghoreishy M. (2016). XNBR-grafted halloysite nanotube core-shell as a potential compatibilizer for immiscible polymer systems. Appl. Surf. Sci..

[9] Hayeemasae N., Sensem Z., Sahakaro K., Ismail H. (2020). Maleated Natural Rubber/Halloysite Nanotubes Composites. Processes.

[10] Hayeemasae N., Sensem Z., Surya I., Sahakaro K., Ismail H. (2020). Synergistic Effect of Maleated Natural Rubber and Modified Palm Stearin as Dual Compatibilizers in Composites based on Natural Rubber and Halloysite Nanotubes. Polymers.

[11] Khunova V., Kristóf J., Kelnar I., Dybal J. (2013). The effect of halloysite modification combined with in situ matrix modifications on the structure and properties of polypropylene/halloysite nanocomposites. Exp. Polym. Lett..

[12] Nicolini K.P., Fukamachi C.R.B., Wypych F., Mangrich A.S. (2009). Dehydrated halloysite intercalated mechanochemically with urea: Thermal behavior and structural aspects. J. Colloid Interface Sci..

[13] Fukamachi C.R.B., Wypych F., Mangrich A. (2007). Use of Fe^3+^ ion probe to study the stability of urea-intercalated kaolinite by electron paramagnetic resonance. J. Colloid Interface Sci..

[14] Trabelsi S., Albouy P.-A., Rault J. (2004). Stress-induced crystallization properties of natural and synthetic cis-polyisoprene. Rubber Chem. Technol..

[15] Toki S., Hsiao B.S. (2003). Nature of strain-induced structures in natural and synthetic rubbers under stretching. Macromolecules.

[16] Candau N., Chazeau L., Chenal J.-M., Gauthier C., Munch E. (2016). A comparison of the abilities of natural rubber (NR) and synthetic polyisoprene cis-1, 4 rubber (IR) to crystallize under strain at high strain rates. Phys. Chem. Chem. Phys..

[17] Lake G.J. (1995). Fatigue and Fracture of Elastomers. Rubber Chem. Technol..

[18] Huneau B. (2011). Strain-induced crystallization of natural rubber: A review of x-ray diffraction investigations. Rubber Chem. Technol..

[19] Tosaka M., Murakami S., Poompradub S., Kohjiya S., Ikeda Y., Toki S., Sics I., Hsiao B.S. (2004). Orientation and Crystallization of Natural Rubber Network As Revealed by WAXD Using Synchrotron Radiation. Macromolecules.

[20] Toki S., Sics I., Ran S., Liu L., Hsiao B.S., Murakami S., Tosaka M., Kohjiya S., Poompradub S., Ikeda Y. (2004). Strain-Induced Molecular Orientation and Crystallization in Natural and Synthetic Rubbers under Uniaxial Deformation by In-situ Synchrotron X-ray Study. Rubber Chem. Technol..

[21] Imbernon L., Pauchet R., Pire M., Albouy P.-A., Tencé-Girault S., Norvez S. (2016). Strain-induced crystallization in sustainably crosslinked epoxidized natural rubber. Polymer.

[22] Poompradub S., Tosaka M., Kohjiya S., Ikeda Y., Toki S., Sics I., Hsiao B.S. (2005). Mechanism of strain-induced crystallization in filled and unfilled natural rubber vulcanizates. J. Appl. Phys..

[23] Chenal J.-M., Gauthier C., Chazeau L., Guy L., Bomal Y. (2007). Parameters governing strain induced crystallization in filled natural rubber. Polymer.

[24] Candau N., Oguz O., Federico C.E., Stoclet G., Tahon J.-F., Maspoch M.L. (2021). Strain induced crystallization in vulcanized natural rubber containing ground tire rubber particles with reinforcement and nucleation abilities. Polym. Test..

[25] Flory P.J., Rehner J. (1943). Statistical mechanics of cross-linked polymer networks I. Rubberlike elasticity. J. Chem. Phys..

[26] Marykutty C., Mathew G., Mathew E., Thomas S. (2003). Studies on novel binary accelerator system in sulfur vulcanization of natural rubber. J. Appl. Polym. Sci..

[27] Osaka N., Kato M., Saito H. (2013). Mechanical properties and network structure of phenol resin crosslinked hydrogenated acrylonitrile-butadiene rubber. J. Appl. Polym. Sci..

[28] Ran S., Zong X., Fang D., Hsiao B.S., Chu B., Phillips R.A. (2001). Structural and morphological studies of isotactic polypropylene fibers during heat/draw deformation by in-situ synchrotron SAXS/WAXD. Macromolecules.

[29] Surya I., Ismail H., Azura A. (2013). Alkanolamide as an accelerator, filler-dispersant and a plasticizer in silica-filled natural rubber compounds. Polym. Test..

[30] Coran A. (2003). Chemistry of the vulcanization and protection of elastomers: A review of the achievements. J. Appl. Polym. Sci..

[31] Piasek Z., Urbanski T. (1962). The infra-red absorption spectrum and structure of urea. Bull. L’Acadeie Pol. Sci. Sér. Sci. Chim..

[32] Horváth E., Kristóf J., Kurdi R., Makó É., Khunová V. (2011). Study of urea intercalation into halloysite by thermoanalytical and spectroscopic techniques. J. Therm. Anal. Calorim..

[33] Makó É., Kristóf J., Horváth E., Vágvölgyi V. (2008). Kaolinite–urea complexes obtained by mechanochemical and aqueous suspension techniques-a comparative study. J. Colloid Interface Sci..

[34] Jia Z., Luo Y., Yang S., Du M., Guo B., Jia D. (2011). Styrene-butadiene rubber/halloysite nanotubes composites modified by epoxidized natural rubber. J. Nanosci. Nanotechnol..

[35] Kadi S., Lellou S., Marouf-Khelifa K., Schott J., Batonneau-Gener I., Khelifa A. (2012). Preparation, characterisation and application of thermally treated Algerian halloysite. Microporous Mesoporous Mater..

[36] Tan W.L., Salehabadi A., Mohd Isa M.H., Abu Bakar M., Abu Bakar N.H.H. (2016). Synthesis and physicochemical characterization of organomodified halloysite/epoxidized natural rubber nanocomposites: A potential flame-resistant adhesive. J. Mater. Sci..

[37] Yuan P., Tan D., Annabi-Bergaya F. (2015). Properties and applications of halloysite nanotubes: Recent research advances and future prospects. Appl. Clay Sci..

[38] Ismail H., Pasbakhsh P., Ahmad Fauzi M.N., Abu Bakar A. (2009). The effect of halloysite nanotubes as a novel nanofiller on curing behaviour, mechanical and microstructural properties of ethylene propylene diene monomer (EPDM) nanocomposites. Polym. Plast. Technol. Eng..

[39] Pasbakhsh P., Ismail H., Ahmad Fauzi M.N., Abu Bakar A. (2010). EPDM/modified halloysite nanocomposites. Appl. Clay Sci..

[40] Payne A.R., Whittaker R.E. (1971). Low strain dynamic properties of filled rubbers. Rubber Chem. Technol..

[41] Nun-anan P., Wisunthorn S., Pichaiyut S., Nathaworn C.D., Nakason C. (2020). Influence of nonrubber components on properties of unvulcanized natural rubber. Polym. Adv. Technol..

[42] Saramolee P., Lopattananon N., Sahakaro K. (2014). Preparation and some properties of modified natural rubber bearing grafted poly (methyl methacrylate) and epoxide groups. Eur. Polym. J..

[43] Kuang W., Yang Z., Tang Z., Guo B. (2016). Wrapping of polyrhodanine onto tubular clay and its prominent effects on the reinforcement of the clay for rubber. Compos. Part A Appl. Sci. Manuf..

[44] Hernández M., López-Manchado M.A., Sanz A., Nogales A., Ezquerra T.A. (2011). Effects of strain-induced crystallization on the segmental dynamics of vulcanized natural rubber. Macromolecules.

[45] Ozbas B., Toki S., Hsiao B.S., Chu B., Register R.A., Aksay I.A., Prud’homme R.K., Adamson D.H. (2012). Strain-induced crystallization and mechanical properties of functionalized graphene sheet-filled natural rubber. J. Polym. Sci. Part B Polym. Phys..

[46] White J.L., Spruiell J.E. (1983). The specification of orientation and its development in polymer processing. Polym. Eng. Sci..

